# Epidemiologie der Virushepatitiden A bis E in Deutschland

**DOI:** 10.1007/s00103-021-03478-8

**Published:** 2022-01-14

**Authors:** Sandra Dudareva, Mirko Faber, Ruth Zimmermann, C.-Thomas Bock, Ruth Offergeld, Gyde Steffen, Julia Enkelmann

**Affiliations:** 1 Abteilung für Infektionsepidemiologie, Robert Koch-Institut, Berlin, Deutschland; 2 Abteilung für Infektionskrankheiten, Robert Koch-Institut, Berlin, Deutschland

**Keywords:** Hepatitis A, Hepatitis B, Hepatitis C, Hepatitis D, Hepatitis E, Epidemiologie, Deutschland, Hepatitis A, Hepatitis B, Hepatitis C, Hepatitis D, Hepatitis E, Epidemiology, Germany

## Abstract

Mit Virushepatitis A bis E werden verschiedene infektiöse Entzündungen des Leberparenchyms bezeichnet, die durch die Hepatitisviren A bis E (HAV, HBV, HCV, HDV und HEV) ausgelöst werden. Zwar ähneln sich die Krankheitsbilder, die Erreger gehören jedoch zu verschiedenen Virusfamilien und unterscheiden sich bezüglich der Pathogenese, der Übertragungswege, des klinischen Verlaufs und der Präventions- und Therapiemöglichkeiten. In Deutschland besteht eine namentliche Meldepflicht nach Infektionsschutzgesetz (IfSG) für den direkten oder indirekten Nachweis und für Verdacht, Erkrankung und Tod. Die Daten werden an das Robert Koch-Institut übermittelt.

In diesem Beitrag wird die Epidemiologie der Hepatitiden A bis E anhand publizierter Studien und Meldedaten beschrieben und es werden aktuelle Herausforderungen und Präventionsansätze aufgezeigt. Letztere bestehen insbesondere in der verbesserten Umsetzung bereits bestehender Impfempfehlungen (Hepatitis A und B), dem verbesserten Zugang zu Prävention, Testung und Versorgung, einschließlich Therapie mit antiviralen Medikamenten (Hepatitis B, C und D), und der Erkennung und Verhinderung lebensmittelbedingter Infektionen und Ausbrüche und Verbesserungen auf dem Gebiet der Lebensmittelsicherheit (Hepatitis A und E).

## Abstract (en)

Viral hepatitis A to E describes various infectious inflammations of the liver parenchyma that are caused by the hepatitis viruses A to E (HAV, HBV, HCV, HDV, and HEV). Although the clinical pictures are similar, the pathogens belong to different virus families and differ in terms of pathogenesis, transmission routes, clinical course, prevention, and therapy options. In Germany, there is mandatory reporting according to the Infection Protection Act (IfSG) for direct or indirect laboratory evidence and for suspicion, illness, and death of viral hepatitis. The data are transmitted to the Robert Koch Institute.

In this article, on the basis of published studies and notification data, we describe the epidemiology of hepatitis A to E as well as current challenges and prevention approaches. In particular, the latter contains the improvement of existing vaccination recommendations (hepatitis A and B); improvement of access to prevention, testing, and care including therapy with antiviral drugs (hepatitis B, C, and D) and the detection and prevention of foodborne infections and outbreaks; and improvements in the field of food safety (hepatitis A and E).

## Einleitung

Mit Virushepatitis A bis E werden verschiedene infektiöse Entzündungen des Leberparenchyms bezeichnet, die durch die Hepatitisviren A bis E (HAV, HBV, HCV, HDV und HEV) ausgelöst werden. Zwar ähneln sich die Krankheitsbilder, die Erreger gehören jedoch zu verschiedenen Virusfamilien und unterscheiden sich bezüglich der Pathogenese, der Übertragungswege, des klinischen Verlaufs und der Präventions- und Therapiemöglichkeiten.

In Deutschland besteht eine namentliche Meldepflicht nach Infektionsschutzgesetz (IfSG) für den direkten oder indirekten Nachweis einer HAV-, HBV-, HCV-, HDV- und HEV-Infektion gemäß § 7 („Labormeldepflicht“) bzw. für Verdacht, Erkrankung und Tod an viralen Hepatitiden gemäß § 6 („Arztmeldepflicht“). Fallmeldungen werden ohne Angabe personenbezogener Daten über die zuständige Landesstelle an das Robert Koch-Institut (RKI) übermittelt.

Dieser Bericht fasst die wichtigsten epidemiologischen Aspekte der Virushepatitiden A bis E in Deutschland anhand von Studien und Meldedaten zusammen. Dabei wird die Inzidenz als Inzidenz der übermittelten Fälle verstanden.

## Hepatitis A

Die Infektion mit dem HAV verursacht bei den meisten Erwachsenen (> 70 %) eine akute symptomatische Hepatitis, wobei die Krankheitsschwere und das Risiko eines fulminanten Verlaufs mit dem Lebensalter und Vorerkrankungen der Leber zunimmt. Bei Kindern verläuft die Infektion meist asymptomatisch oder mit leichten Symptomen [1]. Chronische Infektionen kommen nicht vor, wobei Rückfälle oder protrahierte Verläufe bei bis zu 10–15 % der Personen mit manifester Hepatitis A beschrieben werden [2].

Infizierte Personen scheiden Hepatitis-A-Viren mit dem Stuhl aus. Die Viren sind sehr widerstandsfähig und können über Wochen bis Monate in der Umwelt infektiös bleiben [3]. Die Übertragung erfolgt fäkal-oral, entweder durch den Verzehr von kontaminierten Lebensmitteln und Wasser, direkten Kontakt, Schmierinfektionen z. B. über kontaminierte Gegenstände oder Sexualkontakte. Aufgrund der langen Inkubationszeit (15 bis 50 Tage) und dadurch, dass infizierte Personen bereits 1–2 Wochen vor Symptombeginn ansteckend sind, verlaufen Ausbrüche häufig protrahiert und sind schwer zu kontrollieren.

Eine wirksame Schutzimpfung steht zur Verfügung und wird von der Ständigen Impfkommission (STIKO) empfohlen für alle Reisenden in Regionen mit hoher Hepatitis-A-Inzidenz sowie für Personen mit erhöhtem beruflichen und sexuellen Expositionsrisiko (z. B. Männer, die Sex mit Männern haben – MSM), mit häufigem Kontakt zu Blutbestandteilen, bestehenden Lebererkrankungen und für die Bewohnerschaft psychiatrischer oder vergleichbarer Fürsorgeeinrichtungen. Eine postexpositionelle Immunisierung ist ebenfalls möglich und wird von der STIKO mit einem monovalenten Hepatitis-A-Impfstoff bis 14 Tage nach Kontakt zu Hepatitis-A-Kranken (insbesondere in Gemeinschaftseinrichtungen) empfohlen [4].

Deutschland ist ein Niedriginzidenzland für die Hepatitis A. In der repräsentativen seroepidemiologischen Studie unter Kindern und Jugendlichen in Deutschland 2003–2006 (KiGGS-Studie) wiesen 14 % Antikörper (Anti-HAV) auf; davon waren 11 % mindestens einmal gegen Hepatitis A geimpft, bei den verbleibenden 3 % deuten die Antikörper auf eine durchgemachte Hepatitis-A-Infektion hin. Kinder und Jugendliche mit Migrationshintergrund waren häufiger von Hepatitis-A-Infektionen betroffen, auch wenn sie in Deutschland geboren wurden [5]. In der repräsentativen seroepidemiologischen Studie unter 2008–2011 in Deutschland lebenden Erwachsenen (DEGS-Studie) wurden bei etwa der Hälfte der Männer und Frauen Hepatitis-A-Antikörper nachgewiesen [6]. Wie auch in der Vorgängerstudie 1998 stiegen die Antikörperprävalenzen insgesamt mit dem Alter an. Jedoch wurde 2008–2011 eine deutliche Zunahme der Antikörperprävalenz bei den unter 40-Jährigen beobachtet (vermutlich durch Impfungen z. B. vor Reisen), während in der Altersgruppe der über 50-Jährigen der Anteil von Personen mit Immunität gegen Hepatitis A deutlich zurückging. Personen mit niedrigem sozioökonomischen Status (SES) erwerben ihre Immunität häufiger durch eine Infektion und Personen mit hohem SES häufiger durch eine Impfung [6, 7].

**Figure 1:**
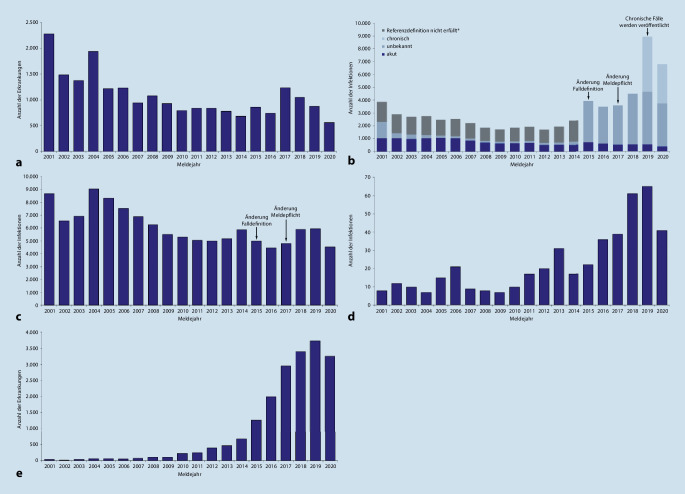

Seit 2001 hat die Zahl der gemeldeten Hepatitis-A-Fälle in Deutschland deutlich abgenommen (Abb. 1a). Wurden im Jahr 2001 noch 2276 Hepatitis-A-Fälle mit erfüllter Referenzdefinition an das RKI übermittelt, waren es 2018–2020 insgesamt 2476; dies entspricht einer mittleren Inzidenz von 1,0 pro 100.000 Einwohnerinnen und Einwohner (Einw.) pro Jahr (Tab. 1). Die Inzidenzen lagen in den letzten 10 Jahren zwischen 0,7 pro 100.000 Einw. (2020) und 1,5 pro 100.000 Einw. (2017). Während der SARS-CoV-2-Pandemie im Jahr 2020 wurden bei verminderter Reiseaktivität und veränderter Inanspruchnahme von medizinischer Versorgung weniger Hepatitis-A-Fälle übermittelt. Im Jahr 2017 waren die Fallzahlen vor allem im Zusammenhang mit internationalen Ausbrüchen unter MSM erhöht [8].

**Table 1:** 

	Hepatitis A	Hepatitis B	Hepatitis C	Hepatitis D	Hepatitis E
*Durchschnittliche Inzidenz der Jahre 2018–2020 (Anzahl pro 100.000 Einw. pro Jahr)*	1,0	8,1	6,6	0,07	4,2
*Nach Altersgruppe*					
*<* *5*	0,7	0,3	0,4	0,00	0,1
*5–9*	1,2	0,4	0,2	0,00	0,1
*10–14*	1,2	0,5	0,1	0,00	0,3
*15–19*	1,0	4,3	0,8	0,01	1,5
*20–24*	1,1	8,6	3,3	0,03	2,2
*25–29*	1,3	13,7	6,4	0,12	2,7
*30–39*	0,8	17,4	13,7	0,16	3,7
*40–49*	0,8	13,4	13,2	0,16	5,1
*50–59*	0,9	8,0	7,6	0,07	6,8
*60–69*	1,0	6,5	5,5	0,04	7,0
*70–79*	1,1	4,1	3,3	0,01	5,2
*>* *79*	1,3	1,9	4,0	0,01	2,9
*Nach Geschlecht*					
*Weiblich*	1,0	6,5	4,0	0,05	3,5
*Männlich*	1,0	9,6	9,2	0,09	4,9
*Im Ausland erworben, Anteil Fälle*^*a*^	32 %	42 %	19 %	46 %	12 %
*Häufigste Nennungen möglicher nichtdeutscher Infektionsländer*^*b*^* (Top 10)*	1. Marokko (12,1 %)	1. Rumänien (8,9 %)	1. Russische Föderation (9,5 %)	k. A.	1. Spanien (13,4 %)
	2. Ägypten (6,6 %)	2. Türkei (8,6 %)	2. Georgien (7,9 %)		2. Italien (7,3 %)
	3. Rumänien (6,0 %)	3. Syrien (6,3 %)	3. Kasachstan (6,6 %)		3. Frankreich (5,9 %)
	4. Spanien (6,0 %)	4. Vietnam (4,4 %)	4. Ukraine (5,8 %)		4. Ägypten (5,5 %)
	5. Türkei (5,9 %)	5. Nigeria (3,9 %)	5. Rumänien (5,3 %)		5. Türkei (4,9 %)
	6. Pakistan (5,7 %)	6. Kasachstan (3,5 %)	6. Weißrussland (4,9 %)		6. Kroatien (4,7 %)
	7. Indien (5,2 %)	7. China (3,5 %)	7. Polen (4,5 %)		7. Griechenland (4,6 %)
	8. Italien (5,0 %)	8. Russische Föderation (3,4 %)	8. Pakistan (4,0 %)		8. Österreich (4,0 %)
	9. Afghanistan (3,0 %)	9. Afghanistan (3,1 %)	9. Italien (3,0 %)		9. Thailand (3,2 %)
	10. Griechenland (2,5 %)	10. Ghana (2,7 %)	10. Bulgarien (2,9 %)		10. Polen (3,0 %)
*Geburtsland nicht Deutschland, Anteil in %*^*a*^	k. A.	70 %	37 %	k. A.	k. A.
*Häufigste Nennungen nichtdeutscher Geburtsländer*	k. A.	1. Türkei (8,4 %)	1. Russische Föderation (15,0 %)	k. A.	k. A.
		2. Rumänien (6,5 %)	2. Kasachstan (9,7 %)		
		3. Syrien (3,4 %)	3. Polen (7,2 %)		
		4. Vietnam (3,3 %)	4. Georgien (6,4 %)		
		5. Russische Föderation (3,2 %)	5. Rumänien (5,7 %)		
		6. Afghanistan (2,7 %)	6. Italien (5,6 %)		
		7. Bulgarien (2,6 %)	7. Ukraine (4,7 %)		
		8. Polen (2,3 %)	8. Türkei (3,3 %)		
		9. Nigeria (2,3 %)	9. Bosnien (2,7 %)		
		10. Kasachstan (2,2 %)	10. Bulgarien (2,6 %)		

*k.* *A.* keine Angabe

^a^Von Fällen mit Angaben zum wahrscheinlichen Infektionsland bzw. zum Geburtsland

^b^Mehrfachnennungen möglich

Durch das Auftreten von Ausbrüchen in verschiedenen Bevölkerungsgruppen variiert die Alters- und Geschlechterverteilung in den unterschiedlichen Jahren z. T. deutlich. In den letzten 3 Jahren wurden ähnliche Inzidenzen bei Männern und Frauen beobachtet. Die höchsten Inzidenzen wurden in den Altersgruppen der 5‑ bis 29-Jährigen und der über 70-Jährigen beobachtet (Tab. 1).

Von den übermittelten Fällen im Zeitraum 2018–2020 hatten 57 % einen Ikterus, 62 % wurden hospitalisiert und 0,3 % (*n* = 8) verstarben aufgrund der Hepatitis A.

Die meisten Hepatitis-A-Erkrankungen wurden in Deutschland erworben. Trotz der bestehenden Impfempfehlung für Reisende in Hepatitis-A-Endemiegebiete und Kostenübernahme durch die meisten Krankenkassen waren 32 % aller Meldefälle, für die Angaben zu möglichen Infektionsländern vorlagen, und 22 % von allen übermittelten Fällen (einschließlich solcher ohne Angaben) reiseassoziiert. Die am häufigsten genannten wahrscheinlichen Infektionsländer waren Marokko, Ägypten, Rumänien, Spanien, die Türkei, Pakistan und Indien (Tab. 1).

Neben vereinzelten lokalen Geschehen, z. B. ausgehend von Gemeinschaftseinrichtungen oder Bäckereien, betrafen Ausbrüche in den letzten Jahren u. a. Asylsuchende aus Endemiegebieten, die in Massenunterkünften untergebracht wurden [9], MSM [10], Reiserückkehrer aus endemischen Gebieten sowie nichtgereiste Personen, die importierte Lebensmittel verzehrt hatten [11, 12].

In Niedriginzidenzländern einschließlich Deutschland werden zunehmend Ausbrüche durch mit HAV kontaminierte Lebensmittel festgestellt. Häufig handelt es sich bei den Vehikeln um Datteln oder gefrorene Früchte, insbesondere Beeren, die aus Endemiegebieten importiert wurden und daraus hergestellte Produkte wie Smoothies, Kuchen/Torten oder Desserts [12–14].

Lebensmittelbedingte Ausbrüche sind häufig überregionale Geschehen und können in der Regel nur mithilfe molekularer Typisierungsverfahren detektiert und epidemiologisch untersucht werden. Aufgrund ungleichmäßiger Kontamination von Lebensmitteln, niedriger Viruslast und schwieriger Probenmatrix gelingt der Polymerasekettenreaktion-(PCR-)Nachweis von Hepatitis-A-Viren in Lebensmitteln häufig nicht [15]. Der epidemiologischen Evidenz kommt daher bei der Untersuchung und Aufklärung solcher Ausbrüche eine wichtige Rolle zu.

## Hepatitis B

HBV kann eine akute, klinische oder subklinische Infektion verursachen, die später in eine chronische Verlaufsform übergehen kann. Eine chronische Verlaufsform liegt dann vor, wenn das HBV-Oberflächenantigen (HBsAg) über mehr als 6 Monate nachweisbar ist. Häufig verläuft die akute Hepatitis B mit unspezifischen Krankheitszeichen.

Nur in etwa einem Drittel der Fälle entsteht das klinische Bild einer akuten ikterischen Hepatitis. In 0,5–1 % der Fälle verläuft die Infektion fulminant mit akutem Leberversagen. Bei bis zu 5 % der HBV-infizierten Erwachsenen entwickelt sich eine chronische Verlaufsform [16]. Bei Kindern erfolgt die Chronifizierung viel häufiger. Eine Infektion im Säuglingsalter führt in 90 % zu einer chronischen Infektion [16]. Etwa 10–20 % der chronisch HBV-infizierten Personen entwickeln eine Zirrhose [16], die zum hepatozellulärem Karzinom (HCC) führen kann.

Eine wirksame Impfung steht zur Verfügung und ist seit 1995 Bestandteil der empfohlenen Standardimpfungen für Säuglinge, Kinder, Jugendliche und Erwachsene mit bestimmten Indikationen [17]. Jedoch liegt die Impfabdeckung unter Kindern mit 65,8–90,5 % noch unter dem angestrebten Ziel von 95 % [18]. Auch bei Indikationsgruppen liegt sie z. T. sogar unter 60 %, wie z. B. bei MSM [18, 19]. Für die Therapie der HBV stehen abhängig vom Stadium der Erkrankung wirksame Therapieoptionen zur Verfügung, welche die Viruslast senken und Spätfolgen verhindern können [20].

Die Übertragung von HBV erfolgt vorwiegend sexuell und durch Kontakt mit kontaminiertem Blut oder anderen Körperflüssigkeiten (z. B. Sperma und Vaginalsekret). Virusreservoir sind vor allem Personen mit chronischer Infektion sowie Neuinfizierte mit hoher Viruslast [21]. Intravenöser Drogenkonsum sowie Wohngemeinschaft mit Virusträgern gehören ebenso zu den häufigsten Übertragungswegen [16]. Die vertikale Übertragung spielt für Personen, die außerhalb Deutschlands in Ländern mit erhöhter Prävalenz und ohne effektive Prävention von Mutter-Kind-Übertragung geboren sind, eine wichtige Rolle. Seit Anfang der 1970er-Jahre werden Blutspenden in Deutschland auf HBsAg getestet, seit 2006 zusätzlich auf Antikörper gegen das Hepatitis-B-Core-Antigen (Anti-HBc). Durch die zunehmend verbesserte Spenderauswahl und durch die immer sensitivere Testung der Spenden liegt das Restrisiko einer transfusionsassoziierten HBV-Infektion unter 1:500.000 [22]. Plasmaderivate sind durch die effektiven Inaktivierungsschritte HBV-sicher [22].

Deutschland zählt in Bezug auf die Allgemeinbevölkerung zu den HBV-Niedrigprävalenzländern. In bevölkerungsbasierten Studien waren 0,3 % der Erwachsenen (2008–2011) und 0,2 % der Kinder (2003–2006) akut oder chronisch mit HBV infiziert (HBsAg-positiv; [6, 23]). Die Anti-HBc-Prävalenz, welche auf eine akute, chronische oder durchgemachte Infektion hinweist, lag in diesen Studien bei 5,1 % unter Erwachsenen und 0,5 % unter Kindern [6, 23]. In den bevölkerungsbasierten Studien DEGS1 und KiGGS waren Gruppen mit erhöhtem Expositionsrisiko unterrepräsentiert [6, 23]. In den anderen Studien konnten für Personen mit injizierendem Drogengebrauch eine HBsAg-Prävalenz von 1,1 % (Datenerhebung 2011–2014) und eine Anti-HBc-Prävalenz von 25 % nachgewiesen werden [24, 25]. Die HBsAg-Prävalenz lag bei Personen mit Migrationshintergrund in Studien zwischen 2,3 % und 3,9 % [25–27]. Nach Geburtsland stratifizierte Daten zur Hepatitis-B-Prävalenz sind jedoch kaum systematisch verfügbar. Unter HIV-positiven Personen wurde eine HBsAg-Prävalenz von 4,5 % berichtet [28].

Ab 2001 bis 2009 wurde ein Rückgang der gemeldeten HBV-Infektionen beobachtet, der vermutlich auch auf einen verbesserten Impfschutz durch die Einführung der generellen Impfempfehlung für Säuglinge im Jahr 1995 zurückzuführen ist (Abb. 1b). Dieser Trend stagnierte mit geringen Schwankungen zwischen den Jahren 2009 und 2014. Die seit 2015 starke Zunahme der gemeldeten Fallzahlen ist darauf zurückzuführen, dass seither auch ausschließlich labordiagnostisch identifizierte HBV-Infektionen unabhängig vom klinischen Verlauf meldepflichtig sind. Mit der IfSG-Änderung im Jahr 2017 sowie der Änderung der Falldefinition, nach der auch chronische Fälle die Referenzdefinition erfüllen, steigen die berichteten Fallzahlen gesamt weiter an [29, 30]. Im Jahr 2020, vermutlich bedingt durch die SARS-CoV-2-Pandemie, wurden 2148 (24 %) weniger Infektionen in allen Infektionsstadien als im Vorjahr gemeldet [30, 31].

Für die Jahre 2018 bis 2020 wurden insgesamt 20.252 Fälle gemeldet und somit lag die bundesweite Inzidenz im Schnitt bei 8,1 Infektionen pro 100.000 Einw. pro Meldejahr (Tab. 1). Dabei war die Inzidenz der akuten Fälle im Vergleich zu chronischen Fällen und Fällen mit unbekanntem Infektionsstadium eher gering und relativ stabil über die Zeit. Die Inzidenz bei Männern (9,6) war höher als bei Frauen (6,5) und die Altersgruppe 25–49 Jahre (13,4–17,4) war am häufigsten betroffen. Die Inzidenz im Kindesalter (< 15 Jahre) war mit 0,3–0,5 pro 100.000 Einw. insgesamt niedrig. 18 Infektionen im Zeitraum vom 2018–2020 entfielen jedoch auf Kinder im ersten Lebensjahr.

Angaben zum wahrscheinlichen Übertragungsweg liegen in den Meldedaten nur selten vor (4,0 %), dennoch ist die Verteilung der angegebenen Übertragungswege stabil über die Zeit. Die am häufigsten übermittelten Übertragungswege in den letzten 3 Jahren waren Wohngemeinschaft mit einem Hepatitis-B-Virusträger (41,2 %), sexuelle Kontakte (24,5 %) und intravenöser Drogenkonsum (23,0 %).

Im Ausland erworbene HBV-Infektionen tragen erheblich zur Zahl der in Deutschland erfassten HBV-Infektionen bei. Angaben zum Infektionsland lagen im Zeitraum 2018–2020 für 5041 (25 %) und zum Geburtsland für 10.761 (53 %) der insgesamt 20.252 Infektionsfälle vor. Der wahrscheinliche Infektionsort lag bei 42 % der Fälle im Ausland. Ein Geburtsland außerhalb Deutschlands wurde bei 70 % der Fälle erhoben. Die häufigsten Infektions- und Geburtsländer sind in der Tab. 1 genannt.

Auch wenn in den letzten 20 Jahren ein erheblicher Rückgang der Inzidenz zu verzeichnen ist, sind zielgerichtete Präventionsmaßnahmen sowie die Identifizierung von akuten und chronischen Infektionen von großer Bedeutung, um die Anzahl der Neuinfektionen weiter zu senken. Der unter Kindern und definierten Indikationsgruppen immer noch unzureichende Impfschutz [18] sollte vervollständigt werden. Da die Krankheitslast in vulnerablen Gruppen erheblich größer ist, muss der Zugang dieser Gruppen zu zielgerichteten Maßnahmen, wie Information, Impfung, Testung und Therapie, verbessert werden.

## Hepatitis D

Eine Infektion mit dem HDV kann als Koinfektion gemeinsam mit dem HBV oder als eine Superinfektion von chronischen Trägern des HBV auftreten. Der Verlauf einer HBV/HDV-Koinfektion ist dem Verlauf einer HBV-Monoinfektion vergleichbar. Nach einer Superinfektion von HBV-Trägern kommt es in 70–90 % der Fälle zu einem zusätzlichen chronischen Verlauf der HDV-Infektionen.

Das HDV ist ein inkomplettes Virus, welches für die Replikation und die Bildung von infektiösen Partikeln das Hüllenprotein des HBV benötigt. Die Übertragung des HDV erfolgt wie bei HBV sexuell, durch kontaminiertes Blut oder Blutprodukte. Das HDV ist schätzungsweise für 18 % der mit Hepatitis B assoziierten Leberzirrhosen und für 20 % der HCC verantwortlich [32, 33]. Jahrzehntelang stand keine wirksame antivirale Therapie gegen HDV zur Verfügung [32, 34, 35]. Im Juli 2020 erhielt die neue Substanz Hepcludex^1^ eine bedingte Marktzulassung in der Europäischen Union (EU). Weitere Substanzen werden zurzeit in klinischen Studien der Phase II und III erforscht [36, 37].

In Deutschland lag die HDV-Prävalenz bei chronisch mit HBV infizierten Personen zwischen 0 % und 7,4 % [28]. In einer bundesweiten Studie aus 74 hepatologischen Schwerpunktzentren lag die HDV-Prävalenz unter HBV-positiven Personen bei 1,4 % [38].

Für die Jahre 2018 bis 2020 lag die bundesweite Inzidenz im Schnitt unter 0,1 gemeldeten Infektionen pro 100.000 Einw. pro Meldejahr (Tab. 1). Der kontinuierliche Anstieg in den Jahren 2015–2017 ist durch Anpassungen der Falldefinition und der Meldepflicht zu erklären ([30]; Abb. 1d). Auch bei Hepatitis-D-Infektion war die Inzidenz bei Männern höher als bei Frauen (Tab. 1). Die Altersgruppe der 30- bis 49-Jährigen weist die höchste Inzidenz auf (0,16). Für 54 % der Fälle lagen Informationen zum Infektionsland vor, von diesen hatte etwa die Hälfte die Infektion in Deutschland erworben.

Die neuen Therapieoptionen für Hepatitis-D-Erkrankte können Folgekomplikationen effektiv reduzieren. Daher sind die rechtzeitige Diagnose und Behandlung wichtig, um die Schwere der Krankheitsverläufe zu reduzieren.

## Hepatitis C

HCV kann sowohl eine akute als auch eine chronische Hepatitis verursachen. Bei einer Persistenz von HCV über 6 Monate spricht man von einer chronischen Infektion. Klinische Symptome treten in diesem Stadium in ca. 25 % der Fälle auf. In etwa 15–45 % eliminiert der Körper innerhalb der ersten 6 Monate das Virus. In den meisten Fällen (bis zu 85 %) kommt es jedoch zu einer Viruspersistenz und damit zu einer chronischen Infektion. Die chronische HCV-Infektion ist für den Großteil der HCV-bedingten Morbidität und Mortalität verantwortlich, obwohl sie häufig erst bei fortgeschrittenem Leberschaden durch Symptome auffällt [39, 40]. Ohne Behandlung entwickeln 16 % der Erkrankten mit chronischer HCV-Infektion innerhalb von 20 Jahren eine Zirrhose [41] und davon 1–4 % pro Jahr ein hepatozelluläres Karzinom [42]. Zwischen 2014 und 2018 wurden mehrere hochwirksame direkt wirkende antivirale Substanzen (DAA) aus verschiedenen Stoffklassen sowie Kombinationspräparate zur Behandlung der Hepatitis C zugelassen. Seitdem kann diese in bis zu 95 % der Fälle geheilt werden, wodurch auch der Entwicklung von Folgeschäden vorgebeugt wird [43].

HCV wird hauptsätzlich parenteral durch Kontakt mit kontaminiertem Blut, in Deutschland vor allem durch injizierenden Drogenkonsum, übertragen. Seit Einführung der diagnostischen Testung aller Blutprodukte auf HCV im Jahr 1991 ist die nosokomiale Übertragung durch Blutkomponenten in Deutschland kein relevanter Übertragungsweg mehr und auch Übertragungen im Rahmen anderer medizinischer Maßnahmen werden selten berichtet. Im Jahr 2017/2018 kam es allerdings in einer Klinik in Bayern zu einem großen nosokomialen Ausbruch mit Übertragung durch medizinische Injektionen und 59 bestätigten Fällen [44]. Eine sexuelle Übertragung von HCV ist grundsätzlich möglich, vor allem bei Sexualpraktiken, bei denen kleinere Verletzungen der Schleimhäute auftreten. Insbesondere bei MSM kommt es zu HCV-Übertragungen im sexuellen Kontext [45, 46]. Bei HCV-positiven Müttern ist die Wahrscheinlichkeit einer perinatalen Übertragung abhängig von der mütterlichen Viruslast und steigt bei HIV-positiven Müttern deutlich an [47].

In Deutschland liegt die Prävalenz von Antikörpern gegen HCV (Anti-HCV) bei 0,3 % in der erwachsenen Allgemeinbevölkerung, damit ist Deutschland ein Niedrigprävalenzland [6, 28]. In einem vom RKI durchgeführten systematischen Review bewegte sich die Gesamtprävalenz von Anti-HCV in der Allgemeinbevölkerung je nach betrachteter Subgruppe zwischen 0,2 % und 1,9 % [28]. Für Gesundheitspersonal wurde in den eingeschlossenen Studien eine sehr niedrige Prävalenz von 0–0,04 % beobachtet, während sie in anderen Bevölkerungsgruppen deutlich höher lag (Menschen mit Migrationshintergrund: 0,4–1,9 %; Menschen mit HIV: 8,2–10,6 %; Menschen, die Drogen injizieren: 63,0–68,0 %; [28]).

Für die Jahre 2018 bis 2020 wurden insgesamt 16.392 aktive HCV-Infektionen an das RKI übermittelt (Tab. 1). Dabei lag die bundesweite Inzidenz im Schnitt bei 6,6 gemeldeten Infektionen pro 100.000 Einw. pro Meldejahr. Im zeitlichen Verlauf war seit 2005 ein abnehmender Trend der Meldeinzidenz zu verzeichnen, der sich seit 2009 verlangsamt (Abb. 1c). Im Jahr 2014 stieg die Inzidenz leicht an und schwankte dann aufgrund von Änderungen im Meldesystem in den folgenden Jahren. Im Jahr 2020 war die Inzidenz um 28 % geringer als in den Vorjahren 2018 und 2019.

In der männlichen Bevölkerung war die Inzidenz zwischen 2018 und 2020 mit im Schnitt 9,2 Infektionen pro 100.000 Einw. mehr als doppelt so hoch wie in der weiblichen (4,0) Bevölkerung (Tab. 1). 30- bis 49-jährige Männer machten dabei mit im Schnitt 20,4 Infektionen pro 100.000 Einw. jährlich einen erheblichen Anteil an der Gesamtzahl der übermittelten HCV-Infektionen aus. Bei den Frauen war die Inzidenz mit 6,8 Infektionen pro 100.000 Einw. in der Altersgruppe der 30- bis 39-Jährigen am höchsten.

Eine Angabe zum wahrscheinlichen Übertragungsweg lag 2018–2020 bei durchschnittlich 25 % der übermittelten Infektionen (*n* = 4106) vor. Intravenöser Drogengebrauch wurde dabei für 65 % dieser Fälle angegeben. Davon lag bei 5 % der Infektionen die nähere Spezifizierung „I.-v.-Drogenkonsum in Haft“ vor. Für 8 % der Fälle lag eine wahrscheinliche Übertragung durch eine Transfusion von Blutprodukten vor 1992 vor und für 9 % eine Übertragung durch andere medizinische Eingriffe (auch im Ausland). Sexuelle Kontakte zwischen Männern wurde in 6 % und heterosexueller Kontakt mit einem HCV-positiven Partner in 3 % der Fälle mit Angabe zum wahrscheinlichsten Übertragungsweg angegeben. Eine perinatale Übertragung wurde für 0,5 % der Fälle berichtet.

Ebenfalls bei durchschnittlich 23 % der zwischen 2018 und 2020 übermittelten HCV-Infektionen fanden sich Angaben zum wahrscheinlichen Infektionsland, dabei wurde Deutschland in 81 % der Fälle genannt (Tab. 1). Als nichtdeutsche Infektionsländer wurden die Russische Föderation, Georgien und Kasachstan am häufigsten angegeben. Für durchschnittlich 46 % der übermittelten Fälle wurden Angaben zum Geburtsland gemacht, dabei entfielen 63 % auf Deutschland.

Von 2014 bis 2020 wurden in Deutschland insgesamt ~ 76.400 Personen in der gesetzlichen Krankenversicherung mit DAA der 2. Generation behandelt. Ein Peak an verschriebenen Therapieregimen pro Monat war im März 2015 zu beobachten, seitdem ist die Zahl der monatlich verschriebenen Therapieregime kontinuierlich rückläufig [48].

Ein Grund für den deutlichen Abfall übermittelter HCV-Infektionen 2020 im Vergleich zu den beiden Vorjahren ist sicherlich die SARS-CoV-2-Pandemie, die durch die hohe Belastung des Öffentlichen Gesundheitsdienstes auch erhebliche Auswirkungen auf die Erfassung, Meldung und Übermittlung von anderen meldepflichtigen Infektionskrankheiten hatte [31, 48]. Auch waren hepatologische Spezialsprechstunden im ersten Lockdown teilweise geschlossen oder Erkrankte haben diese weniger aufgesucht [49]. Dies könnte zu einer Untererfassung von HCV-Neudiagnosen geführt haben. Der stabil gebliebene Anteil übermittelter akuter HCV-Infektionen (etwa ein Fünftel) deutet darauf hin, dass die tatsächliche Inzidenz der HCV-Infektionen in etwa gleich geblieben ist.

Trotz der sehr großen Fortschritte in der HCV-Therapie sinkt seit 2015 in Deutschland die Anzahl der verordneten Therapieregime und es gibt noch immer eine große Anzahl nicht diagnostizierter und nicht therapierter Infizierter [48, 50]. Insbesondere die Weiterleitung von HCV-positiven Menschen mit aktivem Drogenkonsum in eine antivirale Behandlung ist mit erheblichen Hindernissen und Schwierigkeiten verbunden [51, 52]. Um die Inzidenz und Prävalenz in der am stärksten in Deutschland von Hepatitis C betroffenen Gruppe der Drogengebrauchenden langfristig zu senken, besteht hier, wie auch bei Personen in Haft, Verbesserungsbedarf [53].

## Hepatitis E

Noch vor 10 bis 15 Jahren wurde die Hepatitis E als eine in Deutschland seltene Erkrankung wahrgenommen, die ausschließlich akut verläuft und die sich Personen vorwiegend bei Fernreisen nach Afrika oder Asien zuzogen. Diese Sichtweise hat sich grundlegend geändert: Neben den weiterhin vereinzelt auftretenden importierten Infektionen mit Genotyp 1 und 2 bestimmen heute die Genotypen 3 und 4 die Epidemiologie der Hepatitis E als eine in allen westlichen Industriestaaten häufig vorkommende, lebensmittelbedingte Zoonose [54]. In der Regel stehen die typischen Symptome einer akuten Hepatitis im Vordergrund. Zunehmend werden jedoch auch extrahepatische, insbesondere neurologische Manifestationen wie die neuralgische Schulteramyotrophie, das Guillain-Barré-Syndrom und Enzephalitiden beobachtet [55]. Bei immunsupprimierten Personen, insbesondere Transplantatempfängern, kommen auch chronische Infektionen vor [56].

Die Hauptrisikofaktoren für Infektion bzw. Erkrankung sind mittlerweile gut erforscht. In den Industrieländern werden die Genotypen 3 und 4 hauptsächlich durch den Verzehr von Produkten, die aus dem Fleisch infizierter Haus- und Wildschweine hergestellt werden, auf den Menschen übertragen [57–59]. Hierunter finden sich insbesondere verzehrfertige Produkte, die keinen Erhitzungsschritt durch den Konsumenten vorsehen. Mithilfe molekularer Untersuchungen konnte HEV-RNA in Deutschland in Schweinemastbetrieben [60], in kommerziell erhältlichen Schweinelebern [61] und in > 20 % einer Stichprobe von Leber- bzw. Rohwurst aus dem Handel gefunden werden [62]. Eine Übertragung kann ebenfalls durch den Verzehr filtrierender Organismen (z. B. Schalentiere [63]), durch kontaminierte Blutprodukte [64] oder Umweltkontaminationen (z. B. im Rahmen beruflicher Tätigkeiten) stattfinden. Diese spielen jedoch wahrscheinlich eine untergeordnete Rolle [57]. Risikofaktoren für die Entwicklung einer manifesten Erkrankung oder eines schweren Verlaufs nach relevantem Kontakt mit HEV sind neben einem höheren Lebensalter das Bestehen von Vorerkrankungen, insbesondere der Leber [57, 65]. Infektionen mit Genotyp 1 und 2 werden, ähnlich wie bei Hepatitis A, auf Reisen in Endemiegebieten durch die Aufnahme von kontaminierten Speisen oder Wasser ausgelöst. Direkte Mensch-zu-Mensch-Übertragungen scheinen bei allen Genotypen nicht in relevantem Maße vorzukommen. Weitere, mit dem „klassischen“ HEV der Genotypen 1 bis 4 verwandte Viren können bei einer ganzen Reihe unterschiedlicher Tierspezies nachgewiesen werden. Inwiefern diese für die Epidemiologie der Hepatitis E in Deutschland eine Rolle spielen, ist nicht abschließend geklärt.

Der Bedeutungswechsel der Hepatitis E ist anhand der nach IfSG gemeldeten Fälle nachvollziehbar: Wurden in den ersten Jahren nach Einführung des IfSG (2001–2003) etwa 20–30 überwiegend importierte Fälle pro Jahr gemeldet, so waren es im Jahr 2019 mehr als 3700 klinisch-labordiagnostisch gesicherte Fälle, wovon bei 93,7 % keine Reiseanamnese übermittelt wurde (Abb. 1e und Tab. 1). Die Inzidenz stieg entsprechend von 0,03 auf 3,94 Fälle pro 100.000 Einw. und Jahr. Im Jahr 2020 war, wahrscheinlich bedingt durch Effekte der SARS-CoV-2-Pandemie, ein leichter Rückgang der Fallzahlen zu beobachten.

Akute Hepatitis-E-Erkrankungen betreffen hauptsächlich ältere Erwachsene. Fast drei Viertel der übermittelten Erkrankungen der Jahre 2018–2020 traten in der Altersgruppe der 30- bis 79-Jährigen auf; der Inzidenzgipfel liegt in der 6. Lebensdekade (Tab. 1). Bei Kindern ist die Erkrankung offensichtlich sehr selten. In fast allen, insbesondere den höheren Altersgruppen sind Männer deutlich überrepräsentiert. Zur Inzidenz und Prävalenz der chronischen HEV-Infektion bei Immunsupprimierten stehen im Rahmen der gesetzlichen Meldepflicht keine Daten zur Verfügung. In einer im Jahr 2021 veröffentlichten Metaanalyse wurde die Prävalenz der aktiven HEV-Infektion (RNA-Nachweis) bei Empfängern von Organtransplantationen mit 1,2 % angegeben [66].

Bemerkenswert ist der Kontrast zwischen der Häufigkeit diagnostisch gesicherter, klinischer Hepatitis E und der Seroprävalenz in der Bevölkerung. Untersuchungen zweier bevölkerungsrepräsentativer Stichproben der erwachsenen Bevölkerung in Deutschland ergaben eine mit dem Lebensalter zunehmende Prävalenz spezifischer Antikörper (HEV-IgG) von insgesamt 15,3 % (2010) bzw. 18,6 % (1998). Modellierungen legen nahe, dass bereits zwischen 1998 und 2010 jährlich etwa 350.000–500.000 (etwa 5,2 pro 1000 Einw.) neue Infektionen (Serokonversionen) pro Jahr in Deutschland auftraten [67]. In Blutspenden wurde im Jahr 2018 eine Prävalenz der Virämie von 1,68 pro 1000 Spenden (1:597) ermittelt [68]. Die Diskrepanz zu den gemeldeten Fällen kann zum Teil durch den geringen Manifestationsindex der Hepatitis E erklärt werden: Wahrscheinlich verlaufen mehr als 90 % der Infektionen asymptomatisch oder mit lediglich milden Krankheitszeichen und heilen bei immunkompetenten Personen folgenlos aus. Insofern repräsentieren die hierzulande diagnostizierten Infektionen nur die sprichwörtliche Spitze des Eisbergs der (überwiegend alimentären) Exposition der Bevölkerung gegenüber dem HEV. Transiente Virämien im Rahmen asymptomatischer Infektionen bei blutspendenden Personen stellen jedoch auch eine Gefahr für Immunsupprimierte dar, da sich bei diesen nach Empfang der Blutprodukte chronische HEV-Infektionen entwickeln können [69]. Seit 2020 werden deshalb alle Spenden, die für die Herstellung nicht virusinaktivierter Blutprodukte verwendet werden, auf HEV-RNA untersucht.

Die akute Hepatitis E bedarf bei immunkompetenten Personen in der Regel keiner oder allenfalls einer symptomatischen Behandlung. Eine Schutzimpfung ist bislang lediglich in China zugelassen und erhältlich. Eine spezifische, gegen die Hepatitis E gerichtete, medikamentöse Therapie steht nicht zur Verfügung. Insofern kommt der Prävention von Hepatitis-E-Infektionen durch Maßnahmen vonseiten der Lebensmittelsicherheit besondere Bedeutung zu. Bei chronischen Infektionen und fulminanten Verläufen besteht die Möglichkeit eines Therapieversuchs mit antiviralen Medikamenten (z. B. Ribavirin) und pegyliertem Interferon-2-Alpha [70].

## Fazit

Die viralen Hepatitiden A, B, C, D und E haben trotz großer Fortschritte in den letzten Jahren bei der Prävention und Behandlung weiterhin eine große Bedeutung für die Gesundheit der Bevölkerung. Um die globalen Ziele einer Eliminierung von Hepatitis B und C im Sinne einer deutlichen Senkung der Inzidenz und Mortalität [71] zu erreichen, sind weitere Anstrengungen in der Prävention und Behandlung notwendig.

Bei der Hepatitis B muss die Impfabdeckung sowohl bei Kindern als auch in Indikationsgruppen weiter erhöht werden. Dennoch wird es auch bei einer ausreichend hohen Impfquote bei Kindern noch Jahrzehnte dauern, bis die Bevölkerung in Deutschland zum Großteil geschützt ist. Darüber hinaus werden, z. B. durch Migration, kontinuierlich suszeptible Individuen hinzukommen. Das Auffinden der Infektionen und die zum Teil lebenslange Therapie werden deshalb weiterhin eine wesentliche Rolle für die Eliminierung von Hepatitis B spielen. Durch den Schutz vor HBV wird auch ein Schutz vor HDV erzielt. Zurzeit gibt es aber noch keine Möglichkeiten zum Schutz vor einer Superinfektion von HBV-Trägern mit HDV.

Bezüglich der Hepatitis C ist eine einfach zu handhabende, nebenwirkungsarme und hocheffektive Therapie verfügbar. Jedoch wird auch in Deutschland noch ein erheblicher Teil der HCV-infizierten Personen nicht von Therapieangeboten erreicht. Hier gilt es, durch Aufklärung Barrieren sowohl bei Patientinnen und Patienten als auch bei Ärztinnen und Ärzten abzubauen und den Zugang zur Versorgung insbesondere für vulnerable und schwer zugängliche Gruppen zu erleichtern [51, 52]. Darüber hinaus ist eine breit angelegte und auf unterschiedliche Bevölkerungsgruppen angepasste Teststrategie für die Hepatitis-B-, C‑ und -D-Fallfindung essenziell. Ein erster Schritt ist die aktuelle Einführung eines einmaligen kostenlosen Hepatitis-B- und -C-Screenings im Rahmen des medizinischen „Check-up 35“ für Personen mit gesetzlicher Krankenversicherung. Die Elimination von Hepatitis B und C kann in Deutschland nur dann erreicht werden, wenn die Maßnahmen, die in der leicht zugänglichen Allgemeinbevölkerung bereits gut umsetzt sind, auch vulnerablen Gruppen, wie z. B. Personen mit Migrationshintergrund, Drogengebrauchende, Inhaftierte und Wohnungslose, erreichen.

Bezüglich der Hepatitis A und Hepatitis E sehen die AutorInnen aktuelle Herausforderungen insbesondere auf dem Gebiet der Erkennung und Verhinderung lebensmittelbedingter Infektionen und Ausbrüche. Lebensmittel, wie z. B. Fleisch- und Wurstprodukte oder Tiefkühlbeeren, werden heute überwiegend in industriellem Maßstab produziert und überregional vertrieben. Dementsprechend sind Krankheitsfälle, die zu einem lebensmittelbedingten Ausbruch gehören, häufig über ganz Deutschland verstreut und Häufungen ohne die Anwendung molekularer Typisierungsverfahren kaum sichtbar. Der Aufbau einer mit der klassischen Krankheitsüberwachung integrierten molekularen Surveillance ist deshalb von großer Bedeutung. Weiteres Präventionspotenzial besteht in der Elimination oder Reduktion vermehrungsfähiger HAV und HEV in Lebensmitteln, die keine relevanten Erhitzungsschritte durch den Verbraucher vorsehen, der Untersuchung von lebensmittelbedingten Ausbrüchen und des zeitnahen und vollständigen Rückrufs betroffener Produkte. Zudem könnten durch eine noch bessere Umsetzung der Hepatitis-A-Impfempfehlungen Infektionen und Erkrankungen weiter reduziert werden, z. B. unter Reisenden und Personen mit erhöhtem sexuellen Expositionsrisiko.

## Footnotes

1 https://www.ema.europa.eu/en/medicines/human/EPAR/hepcludex.

## References

[1] Hadler SC, Webster HM, Erben JJ, Swanson JE, Maynard JE (1980). Hepatitis A in day-care centers. A community-wide assessment. N Engl J Med.

[2] Glikson M, Galun E, Oren R, Tur-Kaspa R, Shouval D (1992). Relapsing hepatitis A. Review of 14 cases and literature survey. Medicine.

[3] Cook N, Bertrand I, Gantzer C, Pinto RM, Bosch A (2018). Persistence of hepatitis A virus in fresh produce and production environments, and the effect of disinfection procedures: a review. Food Environ Virol.

[4] Ständige Impfkommission (2020). Empfehlungen der Ständigen Impfkommission beim Robert Koch-Institut – 2020/2021. Epidemiol Bull.

[5] Michaelis K, Poethko-Müller C, Kuhnert R, Stark K, Faber M (2018). Hepatitis A virus infections, immunisations and demographic determinants in children and adolescents, Germany. Sci Rep.

[6] Poethko-Müller C, Zimmermann R, Hamouda O, Faber M, Stark K, Ross RS (2013). Die Seroepidemiologie der Hepatitis A, B und C in Deutschland. Bundesgesundheitsblatt Gesundheitsforschung Gesundheitsschutz.

[7] Poethko-Müller C, Schmitz R (2013). Impfstatus von Erwachsenen in Deutschland. Bundesgesundheitsblatt Gesundheitsforschung Gesundheitsschutz.

[8] Zimmermann R, Faber M, Dudareva S, Ingiliz P, Jessen H, Koch J (2021). Hepatitis A outbreak among MSM in Berlin due to low vaccination coverage: epidemiology, management, and successful interventions. Int J Infect Dis.

[9] Michaelis K, Wenzel JJ, Stark K, Faber M (2017). Hepatitis A virus infections and outbreaks in asylum seekers arriving to Germany, September 2015 to March 2016. Emerg Microbes Infect.

[10] Ndumbi P, Freidl GS, Williams CJ, Mardh O, Varela C, Avellon A (2018). Hepatitis A outbreak disproportionately affecting men who have sex with men (MSM) in the European Union and European Economic Area, June 2016 to May 2017. Euro Surveill.

[11] Gassowski M, Michaelis K, Wenzel JJ, Faber M, Figoni J, Mouna L (2018). Two concurrent outbreaks of hepatitis A highlight the risk of infection for non-immune travellers to Morocco, January to June 2018. Euro Surveill.

[12] Ruscher C, Faber M, Werber D, Stark K, Bitzegeio J, Michaelis K (2020). Resurgence of an international hepatitis A outbreak linked to imported frozen strawberries, Germany, 2018 to 2020. Euro Surveill.

[13] Severi E, Verhoef L, Thornton L, Guzman-Herrador BR, Faber M, Sundqvist L (2015). Large and prolonged food-borne multistate hepatitis A outbreak in Europe associated with consumption of frozen berries, 2013 to 2014. Euro Surveill.

[14] Garcia Vilaplana T, Leeman D, Balogun K, Ngui SL, Phipps E, Khan WM (2021). Hepatitis A outbreak associated with consumption of dates, England and Wales, January 2021 to April 2021. Euro Surveill.

[15] Mäde D, Trübner K, Neubert E, Höhne M, Johne R (2013). Detection and typing of norovirus from frozen strawberries involved in a large-scale gastroenteritis outbreak in Germany. Food Environ Virol.

[16] Mauss S, Berg T, Rockstroh J, Sarrazin C, Wedemeyer H (2020). Hepatology—a clinical textbook.

[17] Ständige Impfkommission (2020). Empfehlungen der Ständigen Impfkommission (STIKO) am Robert Koch-Institut 2020/2021. Epidemiol Bull.

[18] Steffen G, Sperle I, Harder T, Sarma N, Beermann S, Thamm R (2021). Hepatitis B vaccination coverage in Germany: systematic review. BMC Infect Dis.

[19] Brandl M, Schmidt A, Marcus U, an der Heiden M, Dudareva S (2020). Are men who have sex with men in Europe protected from hepatitis B?. Epidemiol Infect.

[20] Cornberg MSL, Protzer U, Niederau C, Tacke F, Berg T et al (2021) S3-Leitlinie der Deutschen Gesellschaft für Gastroenterologie, Verdauungs-und Stoffwechselkrankheiten (DGVS) zur Prophylaxe, Diagnostik und Therapie der Hepatitis-B-Virusinfektion. https://www.awmf.org/uploads/tx_szleitlinien/021-011l_S3_Prophylaxe-Diagnostik-Therapie-der-Hepatitis-B-Virusinfektion_2021-07.pdf. Zugegriffen: 19. Okt. 2021

[21] Robert Koch-Institut (2016) Hepatitis B und D RKI Ratgeber. https://www.rki.de/DE/Content/Infekt/EpidBull/Merkblaetter/Ratgeber_HepatitisB.html. Zugegriffen: 19. Okt. 2021

[22] an der Heiden M, Ritter S, Hamouda O, Offergeld R (2015). Estimating the residual risk for HIV, HCV and HBV in different types of platelet concentrates in Germany. Vox Sang.

[23] Cai W, Poethko-Muller C, Hamouda O, Radun D (2011). Hepatitis B virus infections among children and adolescents in Germany: migration background as a risk factor in a low seroprevalence population. Pediatr Infect Dis J.

[24] Haussig JM, Nielsen S, Gassowski M, Bremer V, Marcus U, Wenz B (2018). A large proportion of people who inject drugs are susceptible to hepatitis B: results from a bio-behavioural study in eight German cities. Int J Infect Dis.

[25] Sperle I, Nielsen S, Gassowski M, Naneva Z, Perchemlieva T, Amato-Gauci A (2020). Prevalence of hepatitis C in the adult population of Bulgaria: a pilot study. BMC Res Notes.

[26] Kortas AZ, Polenz J, von Hayek J, Rudiger S, Rottbauer W, Storr U (2017). Screening for infectious diseases among asylum seekers newly arrived in Germany in 2015: a systematic single-centre analysis. Public Health.

[27] Ackermann N, Marosevic D, Hormansdorfer S, Eberle U, Rieder G, Treis B (2018). Screening for infectious diseases among newly arrived asylum seekers, Bavaria, Germany, 2015. Euro Surveill.

[28] Sperle I, Steffen G, Leendertz SA, Sarma N, Beermann S, Thamm R (2020). Prevalence of hepatitis B, C, and D in Germany: results from a scoping review. Front Public Health.

[29] von Laer A, Diercke M, der Heiden AM, Altmann D, Zimmermann R, Dudareva S (2020). Implications of a change in case definition and screening of asylum seekers for hepatitis B surveillance in Germany in 2015 and 2016. Epidemiol Infect.

[30] Burdi S, Harder T, Ullrich A, Krings A, Sandfort M, Dudareva S (2021). Virushepatitis B und D im Jahr 2020. Epidemiol Bull.

[31] Ullrich A, Schranz M, Rexroth U, Hamouda O, Schaade L, Diercke M (2021). Impact of the COVID-19 pandemic and associated non-pharmaceutical interventions on other notifiable infectious diseases in Germany: An analysis of national surveillance data during week 1–2016 – week 32–2020. Lancet Reg Health Eur.

[32] World Health Organization (2018) Hepatitis D. https://www.who.int/news-room/fact-sheets/detail/hepatitis-d. Zugegriffen: 28. Juli 2021

[33] Stockdale AJ, Kreuels B, Henrion MYR, Giorgi E, Kyomuhangi I, de Martel C (2020). The global prevalence of hepatitis D virus infection: systematic review and meta-analysis. J Hepatol.

[34] Shepard CW, Simard EP, Finelli L, Fiore AE, Bell BP (2006). Hepatitis B virus infection: epidemiology and vaccination. Epidemiol Rev.

[35] Taylor JM (2006). Hepatitis delta virus. Virology.

[36] Deterding K, Wedemeyer H (2021). New therapeutic options for hepatitis D. MMW Fortschr Med.

[37] Urban S, Neumann-Haefelin C, Lampertico P (2021). Hepatitis D virus in 2021: virology, immunology and new treatment approaches for a difficult-to-treat disease. Gut.

[38] Fischer C, Mauss S, Zehnter E, Bokemeyer B, Heyne R, Huppe D (2012). Epidemiology and clinical characteristics of patients with chronic hepatitis B (CHB) in Germany—results of a nationwide cross-sectional study. Z Gastroenterol.

[39] Robert Koch-Institut (2018). RKI-Ratgeber Hepatitis C. Epidemiol Bull.

[40] World Health Organization (2019) Hepatitis C. https://www.who.int/news-room/fact-sheets/detail/hepatitis-c. Zugegriffen: 28. Juli 2021

[41] Thein HH, Yi Q, Dore GJ, Krahn MD (2008). Estimation of stage-specific fibrosis progression rates in chronic hepatitis C virus infection: a meta-analysis and meta-regression. Hepatology.

[42] El-Serag HB, Rudolph KL (2007). Hepatocellular carcinoma: epidemiology and molecular carcinogenesis. Gastroenterology.

[43] Sarrazin C, Zimmermann T, Berg T, Hinrichsen H, Mauss S, Wedemeyer H (2020). Addendum: Prophylaxe, Diagnostik und Therapie der Hepatitis-C-Virus(HCV)-Infektion. Z Gastroenterol.

[44] Robert Koch-Institut (2019). Hepatitis-C-Meldedaten nach IfSG, 2016–2018: Auswirkungen der Änderungen von Falldefinition und Meldepflicht. Epidemiol Bull.

[45] Nijmeijer BM, Koopsen J, Schinkel J, Prins M, Geijtenbeek TB (2019). Sexually transmitted hepatitis C virus infections: current trends, and recent advances in understanding the spread in men who have sex with men. J Intern AIDS Soc.

[46] Hoornenborg E, Coyer L, Boyd A, Achterbergh RCA, Schim van der Loeff MF, Bruisten S (2020). High incidence of HCV in HIV-negative men who have sex with men using pre-exposure prophylaxis. J Hepatol.

[47] Benova L, Mohamoud YA, Calvert C, Abu-Raddad LJ (2014). Vertical transmission of hepatitis C virus: systematic review and meta-analysis. Clin Infect Dis.

[48] Meyer E, Steffen G, Krings A, Ullrich A, Kollan C, Dudareva S (2021). Zur Situation bei wichtigen Infektionskrankheiten in Deutschland – Virushepatitis C im Jahr 2020. Epidemiol Bull.

[49] Hüppe D, Niederau C, Serfert Y, Hartmann H, Wedemeyer H (2020). Versorgungsprobleme von Patienten mit chronischer Hepatitis C während der COVID-19-Pandemie und der Lockdown-Verordnungen. Z Gastroenterol.

[50] Zimmermann R, Kollan C, Ingiliz P, Mauss S, Schmidt D, Bremer V (2017). Real-world treatment for chronic hepatitis C infection in Germany: analyses from drug prescription data, 2010–2015. J Hepatol.

[51] Buggisch P, Heiken H, Mauss S, Weber B, Jung M-C, Görne H (2021). Barriers to initiation of hepatitis C virus therapy in Germany: a retrospective, case-controlled study. PLoS ONE.

[52] Gerlich M, Dichtl A, Graf N (2020). Abschlussbericht zum Projekt „HIV? Hepatitis? Das CHECK ich!“.

[53] Oru E, Verster A (2019). Access to hepatitis C care for people who inject drugs and people in prisons. Lancet Gastroenterol Hepatol.

[54] Christou L, Kosmidou M (2013). Hepatitis E virus in the Western world—a pork-related zoonosis. Clin Microbiol Infect.

[55] Jha AK, Kumar G, Dayal VM, Ranjan A, Suchismita A (2021). Neurological manifestations of hepatitis E virus infection: an overview. World J Gastroenterol.

[56] Kamar N, Selves J, Mansuy JM, Ouezzani L, Péron JM, Guitard J (2008). Hepatitis E virus and chronic hepatitis in organ-transplant recipients. N Engl J Med.

[57] Faber M, Askar M, Stark K (2018). Case-control study on risk factors for acute hepatitis E in Germany, 2012 to 2014.

[58] Tulen AD, Vennema H, van Pelt W, Franz E, Hofhuis A (2019). A case-control study into risk factors for acute hepatitis E in the Netherlands, 2015–2017. J Infect.

[59] Said B, Ijaz S, Chand MA, Kafatos G, Tedder R, Morgan D (2014). Hepatitis E virus in England and Wales: indigenous infection is associated with the consumption of processed pork products. Epidemiol Infect.

[60] Baechlein C, Seehusen F, Nathues H, grosse Beilage E, Baumgärtner W, Grummer B (2013). Molecular detection of hepatitis E virus in German domestic pigs. Berl Munch Tierarztl Wochenschr.

[61] Wenzel JJ, Preiss J, Schemmerer M, Huber B, Plentz A, Jilg W (2011). Detection of hepatitis E virus (HEV) from porcine livers in Southeastern Germany and high sequence homology to human HEV isolates. J Clin Virol.

[62] Szabo K, Trojnar E, Anheyer-Behmenburg H, Binder A, Schotte U, Ellerbroek L (2015). Detection of hepatitis E virus RNA in raw sausages and liver sausages from retail in Germany using an optimized method. Int J Food Microbiol.

[63] Said B, Ijaz S, Kafatos G, Booth L, Thomas HL, Walsh A (2009). Hepatitis E outbreak on cruise ship. Emerg Infect Dis.

[64] Bi H, Yang R, Wu C, Xia J (2020). Hepatitis E virus and blood transfusion safety. Epidemiol Infect.

[65] Zhang S, Chen C, Peng J, Li X, Zhang D, Yan J (2017). Investigation of underlying comorbidities as risk factors for symptomatic human hepatitis E virus infection. Aliment Pharmacol Ther.

[66] Buescher G, Ozga AK, Lorenz E, Pischke S, May J, Addo MM (2021). Hepatitis E seroprevalence and viremia rate in immunocompromised patients: a systematic review and meta-analysis. Liver Int.

[67] Faber M, Willrich N, Schemmerer M, Rauh C, Kuhnert R, Stark K (2018). Hepatitis E virus seroprevalence, seroincidence and seroreversion in the German adult population. J Viral Hepat.

[68] Vollmer T, Diekmann J, Knabbe C, Dreier J (2019). Hepatitis E virus blood donor NAT screening: as much as possible or as much as needed?. Transfusion.

[69] Denner J, Pischke S, Steinmann E, Blumel J, Glebe D (2019). Why all blood donations should be tested for hepatitis E virus (HEV). BMC Infect Dis.

[70] Velavan TP, Pallerla SR, Johne R, Todt D, Steinmann E, Schemmerer M (2021). Hepatitis E: an update on One Health and clinical medicine. Liver Int.

[71] World Health Organization (2016) Global health sector strategy on viral hepatitis 2016–2021. Towards ending viral hepatitis. http://apps.who.int/iris/bitstream/10665/246177/1/WHO-HIV-2016.06-eng.pdf?ua=1. Zugegriffen: 28. Juli 2021

